# Potential of nanobiosensor in sustainable agriculture: the state-of-art^☆^

**DOI:** 10.1016/j.heliyon.2022.e12207

**Published:** 2022-12-08

**Authors:** Rittick Mondal, Paulami Dam, Joydeep Chakraborty, Mathew L. Paret, Ahmet Katı, Sevde Altuntas, Ranit Sarkar, Suvankar Ghorai, Debnirmalya Gangopadhyay, Amit Kumar Mandal, Azamal Husen

**Affiliations:** a Chemical Biology Laboratory, Department of Sericulture, Raiganj University, North Dinajpur, West Bengal 733134, India; b Department of Microbiology, Raiganj University, North Dinajpur, West Bengal 733134, India; c North Florida Research and Education Center, Institute of Food and Agricultural Sciences, University of Florida, Quincy, FL 32351, USA; d Plant Pathology Department, Institute of Food and Agricultural Sciences, University of Florida, Gainesville, FL 32611, USA; e Department of Biotechnology, University of Health Sciences Turkey, 34668, Istanbul, Turkey; f Experimental Medicine Research and Application Center, University of Health Sciences Turkey, 34668, Istanbul, Turkey; g Department of Tissue Engineering, University of Health Sciences Turkey, 34668, Istanbul, Turkey; h Department of Microbiology, Orissa University of Agriculture & Technology, Bhubaneswar, Odisha 751003, India; i Silkworm Genetics and Breeding Laboratory, Department of Sericulture, Raiganj University, North Dinajpur, West Bengal 733134, India; j Wolaita Sodo University, PO Box 138, Wolaita, Ethiopia

**Keywords:** Nanotechnology, Conventional farming practices, Pathogens, Detection limit

## Abstract

A rapid surge in world population leads to an increase in worldwide demand for agricultural products. Nanotechnology and its applications in agriculture have appeared as a boon to civilization with enormous potential in transforming conventional farming practices into redefined farming activities. Low-cost portable nanobiosensors are the most effective diagnostic tool for the rapid on-site assessment of plant and soil health including plant biotic and abiotic stress level, nutritional status, presence of hazardous chemicals in soil, etc. to maintain proper farming and crop productivity. Nanobiosensors detect physiological signals and convert them into standardized detectable signals. In order to achieve a reliable sensing analysis, nanoparticles can aid in signal amplification and sensor sensitivity by lowering the detection limit. The high selectivity and sensitivity of nanobiosensors enable early detection and management of targeted abnormalities. This study identifies the types of nanobiosensors according to the target application in agriculture sector.

## 1 Introduction

The agriculture sector is always most significant because it harvests and offers raw materials for food and feed industries and steadily maintains its economic status. Globally, it is expected that around 874 million people (27% of the global workforce) are actively engaged in the agriculture sector (FAO 2021; FAO 2020). Industrialization and the limit of natural resources hinder the extension of the agriculture sector in terms of both quality and quantity. Worldwide around 127 million hectares of the agricultural plot have deteriorated from 2000 to 2019. The global rise of hunger or undernourishment is also increasing drastically every year. Nearly 610 million and 652 million people were reported to be suffered from undernourishment in the years 2014 and 2019 respectively. But these numbers have dramatically increased to 770 million people in 2020. According to the prediction of the researchers, the food importunity is about to accelerate to 98% by 2050 as the global population is expected to reach nearly 10 billion (FAO 2021; Duro et al., 2020; FAO 2020). Rapid industrialization has increased the extensive use of inorganic fertilizers, harmful chemicals such as pesticides, etc. These chemicals have a significant role in the agro ecosystem's disbalance, and serious public health problems (Stamati et al., 2016). In the year 2019, almost 190 million tonnes of inorganic fertilizer have been applied to agricultural land and the use of chemical pesticides has also increased by around 36% from 2000 to 2019 (FAO 2021).

The agroecosystem is also confronted with some other significant difficulties, such as crop diseases, and heavy metal contamination in soil (Sharma et al., 2021). The contaminated or adulterated foodstuffs show some serious harsh effects on the consumers (Schieber 2018). It is of utmost importance to detect and monitor the harmful chemicals, pathogens, metals, etc. in agricultural products for ensuring the safety of foods we consume. Different kinds of sophisticated lab techniques are present including spectroscopy, chromatography, enzymatic methods, proteomics, metabolomics, stable isotope analysis, etc (Schieber 2018; Attrey 2017). Although sophisticated lab techniques are efficient and reliable for detecting unwanted stuff in farming practices and their products, they are tedious, costly, and need more proficient operators. Therefore, developing rapid, reliable, user-friendly, and on-site screening methods is crucial. The advancement of nanotechnology showed great potential to overcome such problems (Konur 2021; Neme et al., 2021). Nanobiosensor is the combination of nanomaterials with biosensors, are sensitive, non-invasive, and specifically designed for the monitoring of a plethora of environmental samples (Thakur et al., 2022; Kalyani et al., 2021). The metal NPs (Au, Ag, etc.), metal oxides NPs (ZnO, TiO_2_, MgO, etc.), magnetic NPs, CNPs, dendrimers, QDs nanoparticles, etc. were used in nanobiosensors for the target detection (Singh 2021). A nanobiosensor comprises of three components i.e. biological probe or recognition elements (such as aptamers, antibodies, peptides, and enzymes), transducer, and detector (Naresh and Lee 2021). In the agricultural sector, these portable easy to use devices has tremendous potential and have been used to on-site monitor the soil and plant physiological conditions such as soil fertility, pH, moisture content, mineral concentrations, level of toxic chemicals, prior detection of pests, and pathogens (Nadporozhskaya et al., 2022; Sharma et al., 2021). Nanobiosensors possess abundant scope in sustainable farming practices due to their sensing ability with a low limit of detections (Mathivanan et al., 2021; Sharma et al., 2021).

Given the aforementioned facts, the present review gives a brief outline of nanobiosensors and their utility in agriculture to monitor soil health, plant growth conditions, and the quality of food grains.

## 2 Methods

This review included studies published between 2016 to 2022, other articles were also incorporated outside the specified period based on the number of citations and relevance of experimental investigations. The present study aimed to discuss and compare the data on different available nano-biosensors from scientific databases like Web of Science, PubMed, Google Scholar, and Scopus. The search terms encompass “nano-biosensor,” “bioreceptor,” “aptamer,” “lateral flow assay,” “gold nanoparticles,” “SWCNTs”, “MWCNTs”, “magnetic nanoparticles”, “CNPs”, “platinum nanoparticles”, “silver nanoparticles,” “plant pathogen,” “abiotic stress,” “chemical pesticides”, “wearable biosensor”.

The added value of this study:

• We overviewed and assessed different nanobiosensor and their types based on their bio-recognition and transducer parts.
• We reported a comprehensive account of the recent trends and practices in the sensing strategies that were implemented in agriculture.
• We reported the selection criteria of aptamers through SELEX technology and its interaction with the counterpart via the formation of secondary structure that includes lateral flow assay mediated pathogen detection process.
• This review also provides a short outline of the smart nanobiosensors i.e. wearable plant sensors and e-monitoring devices used for crop health monitoring.
• We highlighted how nano-priming can be an innovative technique to enhance the fitness of plants exposed to stress conditions.

## 3 Nanobiosensor and its type

A biosensor is a self-contained unified device that employs a bioreceptor exhibiting sensitivity towards the detectable biomolecules i.e. analytes in order to produce fine quantitative analytical data. Herein, physical or chemical transducers are combined with recognition elements for the detection of biological products (Bhalla et al., 2016). The biological layers interact with the analytes, whereas the information from the interaction is converted into a quantifiable impact by the transducers (Huang et al., 2021). The transducers and recognition elements of a biosensor may be used to categorize the biosensor varieties. A biosensor can be electrochemical (amperometric, impedimetric, potentiometric, etc.), calorimetric, piezoelectric (quartz crystal microbalance, acoustic wave, etc.), or optical (reflection, refraction, absorption, transmission, fluorescence, surface plasmon resonance, waveguide, etc.) on the basis of transducers (Malhotra and Ali, 2018). Biosensors can be classified as aptamer-based, enzymatic, nucleic acid-based, oligonucleotide-based, immunosensors (antigen-antibody interaction or biomarker approaches), whole-cell, organelle based, etc. depending on their recognition elements (Malhotra and Ali, 2018). Along with this, some versatile paper-based biosensors (e.g. microfluidics biosensors, lateral flow assays (LFA), and dipstick test) are also present, where the detection of the biomarkers is based on optical detection as well as electrochemical detection, chemiluminescence or electrochemiluminescence detection (Kuswandi and Ensafi 2019). Current investigations are being focused on introducing nanoparticles into biosensor fabrication so that the system's sensitivity and performance can be enhanced.

## 4 Sensors based on the types of transducers

### 4.1 Electrochemical biosensors

In electrochemical biosensors, bioreceptors coated or covalently linked on a transducer surface when interacting with the analytes produce an electrochemical signal in various types of patterns. It enables label-free detection along with a high ratio of signal-to-volume. Thus, this specific type of biosensor performs via potentiometric, impedimetric, amperometric, and capacitive transducers to transform biological or chemical signals into quantifiable means, on the basis of their working principle (Malhotra and Ali, 2018). An amperometric biosensor detects the change in current whereas the potentiometric biosensor measures the potential generated via a chemical reaction when the electroactive molecules interact. Interaction between biomolecules in enzymatic techniques, nucleic acid-based approaches, antigen-antibody interactions, etc. may be quantified using impedimetric and capacitive biosensors (Malhotra and Ali, 2018). Impedimetric biosensor tracks alteration of charge conductance and capacitance as the target binds selectively to the sensor surface (Kim et al., 2019). The class of affinity biosensors which detect direct interaction between the target molecule and the sensor surface includes capacitive biosensors. It evaluates the thickness of the dielectric layers or assesses the changes in the dielectric characteristics at the electrolyte-electrode interface (Ertürk and Mattiasson 2017).

### 4.2 Optical biosensors

Optical biosensors measure changes in optical properties like wavelength, refractive index, intensity, and polarisation to detect biological interactions through label-based and label-free detectors (Huertas et al., 2019). Optical labels are used to detect color changes or the availability of photon particles produced at a certain wavelength representing the presence of analytes in a sample. Label-free optical biosensors allow the conversion of a particular biomolecular interaction into a measurable signal using a variety of techniques, such as light scattering or the production of an evanescent wave (Huertas et al., 2019). In terms of the working principle, optical biosensors are of different types i.e. surface plasmon resonance (SPR)-based, optical waveguide-based, optical resonator-based, photonic crystal-based, and optical fiber-based (Chen and Wang, 2020). For instance, SPR belongs to label-free optical biosensing technologies. The SPR method is based on the optical measurement of refractive index changes associated with the binding of analyte molecules (Azzouz et al., 2022).

### 4.3 Calorimetric biosensors

The literature has demonstrated the utilization of calorimetry as a transduction mechanism in biosensors for the detection of a wide variety of analytes. These biosensors record the heat production (or dissipation) driven by the precise interaction of the analytes with receptors (e.g. substrate and enzyme), correlating it to the analyte concentration. Herein, two distinct types of technologies are frequently used: (1) thermopile-based device, dependent on the Seebeck effect, and (2) thermistor-based device, focused on the variation in resistance with the temperature fluctuation (Vermeir et al., 2007). The sensor typically employs two mechanisms such as adiabatic calorimetry which involves no heat transfer between the reaction vessel and surroundings and heat conduction calorimetry where heat transfers are observed between the vessel and external environmental heat sink (Das et al., 2022). Calorimetric sensors are often utilized in a wide range of sectors, including the biochemical, biomedical, and pharmaceutical industries (Das et al., 2022).

### 4.4 Piezoelectric biosensor

The piezoelectric biosensor is one of the analytical platforms which record affinity interaction on the electrode surface (Pohanha, 2018a). Jacques Curie and Pierre Curie identified that anisotropic crystals (i.e. crystals without a center of symmetry), can produce an electric dipole when physically compressed, leading to the discovery of the piezoelectric phenomenon (Pohanka, 2017). The property of a material to generate voltage under physical stress is referred to as the piezoelectric effect. Two electrodes when providing alternating voltage to the crystal surface create mechanical oscillations, and the frequency is thereby measured in this type of biosensor. The presence of analytes causes the oscillation frequency to change proportionately with the concentration of analytes (Pohanka, 2018b). Assays focused on the interaction between antigen/antibody, polynucleotide strains, aptamer/protein can utilize the piezoelectric biosensors (Pohanka, 2017). Therefore, conventional immunosensing approaches, detecting nucleic acids, sensing microbes, and cellular biology applications are now of utmost importance in the fields of piezoelectric biosensor practices (Skládal, 2016).

## 5 Sensors based on the types of biological recognition elements

On the basis of transducers, the biosensor can be of the above-mentioned types of biosensors. Furthermore, a biosensor must possess a recognition element conjugated with the transducer. Based on the biological recognition element the biosensor can be classified as follows.

### 5.1 Immunosensors

An immunosensor is a kind of biosensor which is based on the conventional antigen-antibody interaction and employs the antibodies as the biological recognition elements of the antigen molecules. The interaction is measured by the transducers and the generated electrical signal is generally quantified (Pérez-Fernández and de la Escosura Muñiz, 2022; Aydin et al., 2019). The immunosensors' key benefits are attributed to the stability, specificity, and sensitivity of antibody molecules (Pérez-Fernández and de la Escosura Muñiz, 2022). Non-labeled immunosensors and labeled immunosensors are categorized as the two types of immunosensors depending on their working principle. In non-labeled sensors, the antigen-antibody complex on the transducer surface is directly detected by determining the physical alterations caused by the immunocomplex formation. A labeled immunosensor, on the other hand, involves a sensitive detectable label and label-based measurement (Lim and Ahmed, 2019).

### 5.2 Enzymatic biosensors

The enzyme is the core component of an enzymatic biosensor. In a third-generation biosensing (direct electron transfer between redox proteins and the electrode) platform, enzyme-based biosensors prioritize the electrochemical transducers during the instrumentation. Hence, enzyme electrodes integrate the enzymes' excellent specificity with the benefits of electrochemical sensing, i.e. high sensitivity, cheap cost, and easy monitoring (Zhao et al., 2017). Herein, enzymes are usually globular proteins comprised of linear amino acid chains which can fold themselves into three-dimensional structures providing the specificity the system requires. Factors such as heat stability and the rapid turnover number are critical for the enzyme bioreceptor in an enzymatic biosensor. Therefore, the ideal candidates for the production of biosensors under such circumstances are enzymes derived from thermophilic bacteria or enzymes that have undergone genetic modification (Verma 2017a). Nevertheless, enzyme immobilization can enhance properties that are essential for biosensor transduction, including surface-to-volume ratio, catalytic activity, sensitivity, selectivity, etc (Cavalcante et al., 2017).

### 5.3 Aptasensors

**Figure 1:**
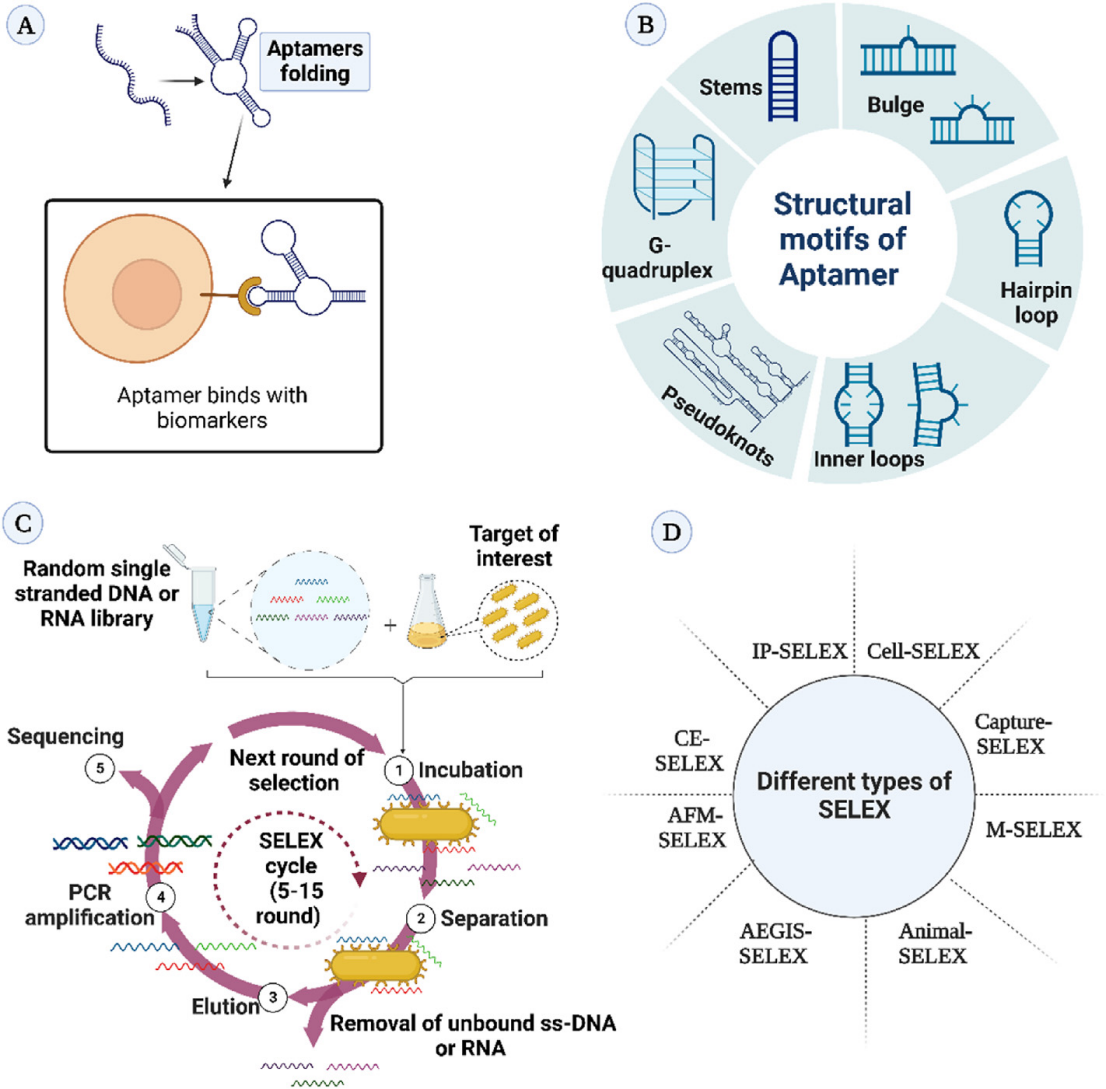
(A) Aptamer binds with its target of interest via non-covalent interactions; (B) Different types of structural motifs of aptamer; (C) Selection of aptamer through the SELEX technique; (D) Various types of SELEX techniques.

Aptamers (single-stranded oligonucleotides or peptide molecules) are used as bioreceptors. Recently aptamers are attracting a lot of interest owing to their superior functionalities (e.g., high stability in the wide ranges of pH and temperatures) and low developing cost. Aptamers are ideally 15–40 bases long and form three-dimensional structures to bind specifically with their complementary region through van der Waals forces, hydrogen bonding, and electrostatic interactions (Figure 1A). Aptamers fold into different types of structural motifs like stems, inner loops, hairpin structures, purine-rich bulges, G-quadruplex structures, kissing complexes, and pseudoknots (Zhao et al., 2018) (Table 1) (Figure 1B). The aptamers are screened through the SELEX process (Figure 1C). Several SELEX-based aptamer selection approaches have been developed, such as whole cell-SELEX, capture-SELEX, immunoprecipitation-coupled SELEX, microfluidic-SELEX, capillary electrophoresis-SELEX, artificially expanded genetic information system-SELEX, animal-SELEX, and atomic force microscopy SELEX (Guo et al., 2020; Zhang et al., 2019) (Figure 1D). As per the study, it is estimated that the value of the aptamer market in 2021 is around 151 million USD, which will increase to 342 million USD by 2026 with a compound annual growth rate of 17.7% amidst the forecast period (https://www.marketsandmarkets.com/Market-Reports/aptamers-technology-market-1167.html; Date-06/08/2022). The hike in the aptamer market is due to the rising demand for aptamer-based detection tools for chronic and rare diseases. Nowadays aptamers have been extensively explored as biorecognition molecules of LFA, which are used as an on-site detection kit. Typically, the LFA contains a sample pad, conjugate pad, nitrocellulose membrane, and absorbent pad. The sample is placed over the sample pad for uniform diffusion and capillary action guides it to the following analytical areas. The sample pad is treated with buffer salts and other active chemicals to maintain the stability and flow rate of the sample. The labeled biorecognition molecule is placed over the conjugate pad. The detection process occurs on the membrane. The absorbent pad prevents the backflow of the loaded sample (Huang et al., 2021; Zhao et al., 2018).

**Table 1:** Aptamer sequences against the specific target of interest and their secondary structure (predicted through the MFold online portal; the portal for the mfold web server is http://www.unafold.org/mfold/applications/dna-folding-form.php; Zuker, 2003).

Specific target	Aptamer sequence	Kd value	Possible secondary structure	References
λ-cyhalothrin	5′-ACCGACCGTGCTGGACTCTAGGGGAAGCACGGGCGGGGAATGCAACACGAGTATGAGCGAGCGTTGCG-3′	50.64 ± 4.33 nmol/L		Yang et al. (2021)
Deoxynivalenol	5′–(SH)–(CH_2_)_6_-GCATCACTACAGTCATTACGCATCGTAGGGGGGATCGTTAAGGAAGTGCCCGGAGGCGGTATCGTGTGAAGTGCTGTCCC-3′	-		Subak et al. (2021)
Diazinon	5′–NH_2_–(CH_2_)_6_-ATCCGTCACACCTGCTCTAATATAGAGGTATTGCTCTTGGACAAGGTACAGGGATGGTGTTGGCTCCCGTAT-3′	-		Talari et al. (2021)
Aflatoxin B1	5′-TGGGGTTTTGGTGGCGGGTGGTGTACG GGCGAGGG-3′	-		Lin and Shen (2020)
Zearalenone	5′–NH_2_–TCATCTATCTATGGTACATTACTATCTGTAATGTGATATG-3′	-		
Ochratoxin A	5′-GCTGAGTCTGAGTCG ATCGGGTGTGGGTGGCGTAA AGGGAGCATCGGACA-3′	-		Jin et al. (2018)
Mercury	5′-GCTGAGTCTGAGTCGTCATGTTTGTTTGTTGGCCCCCCTTCTTTCTTA-3′	-		
Acetamiprid	5′–(SH)–(CH_2_)_6_-TGTAATTTGTCTGCAGCGGTTCTTGATCGCTGACACCATATTATGAAGA-[Flc]-3′	-		Madianos et al. (2018)
Atrazine	5′–(SH)–(CH_2_)_6_-TACTGTTTGCACTGGCGGATTTAGCCAGTCAGTG[Flc]-3′	-		
Chlorpyrifos	5′-CCTGCCACGCTCCGCAAGCTTAGGGTTACGCCTGCAGCGATTCTTGATCGCGCTGCTGGTAATCCTTCTTTAAGCTTGGCACCCGCATCGT-3′	-		Jiao et al. (2017)
Abscisic acid	5′- GCGGATGAAGACTGGTGTGAGGGGATGGGTTAGGTGGAGGTGGTTATTCCGGGAATTCGCCCTAAATACGAGCAAC-3′	-		Wang et al. (2017)

Nevertheless, bioreceptors or biological recognition elements may also include other kinds of biomolecules such as nucleic acids, oligonucleotides, entire cells, cell organelles, and so on. Based on the specific type of bioreceptors the biosensors are thus conventionally named. It further ingrates several transducer approaches and works as a sensing system and detection platform.

## 6 Different nanomaterials for nanobiosensors

Traditional agricultural detection techniques are generally costly and time-consuming approach that necessitates many sample preparation procedures prior to detection, as well as skilled workers and complex instruments that are not easily obtainable, especially for individuals existing in remote areas (Mufamadi and Sekhejane 2017). The use of nanoparticles in the fabrication of biosensors has the ability to conquer the obstacles of older approaches. Some of the benefits of nanoparticle-based biological sensors over conventional methodologies are as follows: They possess excellent sensitivity and specificity, as well as a fast and effective response time with real-time output. Furthermore, the method may detect a single or several analytes that could be dangerous to plants, animals, or humans at extremely low concentrations, using a tiny sample preparation and portable gear (Mufamadi and Sekhejane 2017). In addition, because they may be used in a number of combinations, we might see them being used as a point-of-care system or multiplexed device. Several nanoparticles are currently grabbing the center of attention in biosensing research. Biological probes paired with different nanoparticles such as metallic, quantum dots, magnetic, graphene oxide, and carbon nanotube can detect several analytes (Thakur et al., 2022). Fullerene, titanium, silicon oxide, etc. are some other materials that are occasionally used in this field. Nevertheless, polyacrylic acid, zeolite, and chitosan are a few examples of polymers that can be utilized for encapsulating purposes (Thakur et al., 2022). Due to its versatility, different metal nanoparticles, typically made of gold and silver can be employed for nanobiosensors. It was found that AuNPs are extensively used among noble metals followed by CuNPs, magnetic nanoparticles of Fe_3_O_4,_ etc. Moreover, multiwalled carbon nanotubes/AuNPs, quantum dots upconverting nanoparticles of magnetic beads and cadmium telluride quantum dots/magnetic nanoparticles are some of the hybrid nanomaterials frequently used in studies (Tessaro et al., 2022). The monitoring of sustainable agricultural practices in the areas of food safety and arbitrary input of excessive herbicides is extremely effective using nanobiosensors which permit rapid screening of pollutants in soil and water at nanomolar to picomolar levels (Verma and Rani, 2021).

For the agricultural industry, carbon nanoparticles and zinc oxide nanoparticles are multifunctional nanomaterials due to their exceptional electrical, optical, and chemical characteristics, along with low toxicity and good biocompatibility (Shojaei et al., 2019; Sabir et al., 2014). Noble metal nanoparticles such as gold, platinum, etc. are also being extensively used because of their unique sensing properties (Yu et al., 2021; Lau et al., 2017). The use of selenium nanoparticles in biosensors was also observed in agricultural fields (Ahmed et al., 2020). Moreover, nanoparticle composites such as chitosan/gold nanoparticles-graphene nanosheet, etc. hold great potential in this sector (Bao et al., 2015). Graphene is highly biocompatible and contains oxygenated functional groups, which allow chemical functionalization to form metal composites. Due to these distinctive features, graphene-based biosensors are considered as promising candidates (Verma et al., 2022). Among the uses of nanobiosensors, sensing soil quality, plant pathogens, phytohormones, pesticides, and heavy metals are of utmost importance (Ali et al., 2021). The advancement of an ultrasensitive nanobiosensor requires a deeper understanding of how biomolecules work at the nano-bio interface and the adoption of the optimal bioconjugation method (Verma 2017b).

## 7 Agricultural application of nanobiosensor

In the case of maintaining sustainable farming, new modern technology is required, including nanobiosensor. Nanobiosensors offer a low-cost detection tool with greater sensitivity in agri-food industries. The inclusion of nanoparticles and nanostructures into sensors significantly improves device efficiency in terms of sensitivity, selectivity, multiplexed detection capabilities, and portability (Kumar et al., 2021; Sharma et al., 2021). Some of the potential applications of nanobiosensors are listed below:

### 7.1 Nanobiosensor in stress management

#### 7.1.1 Biotic stress management

**Figure 2:**
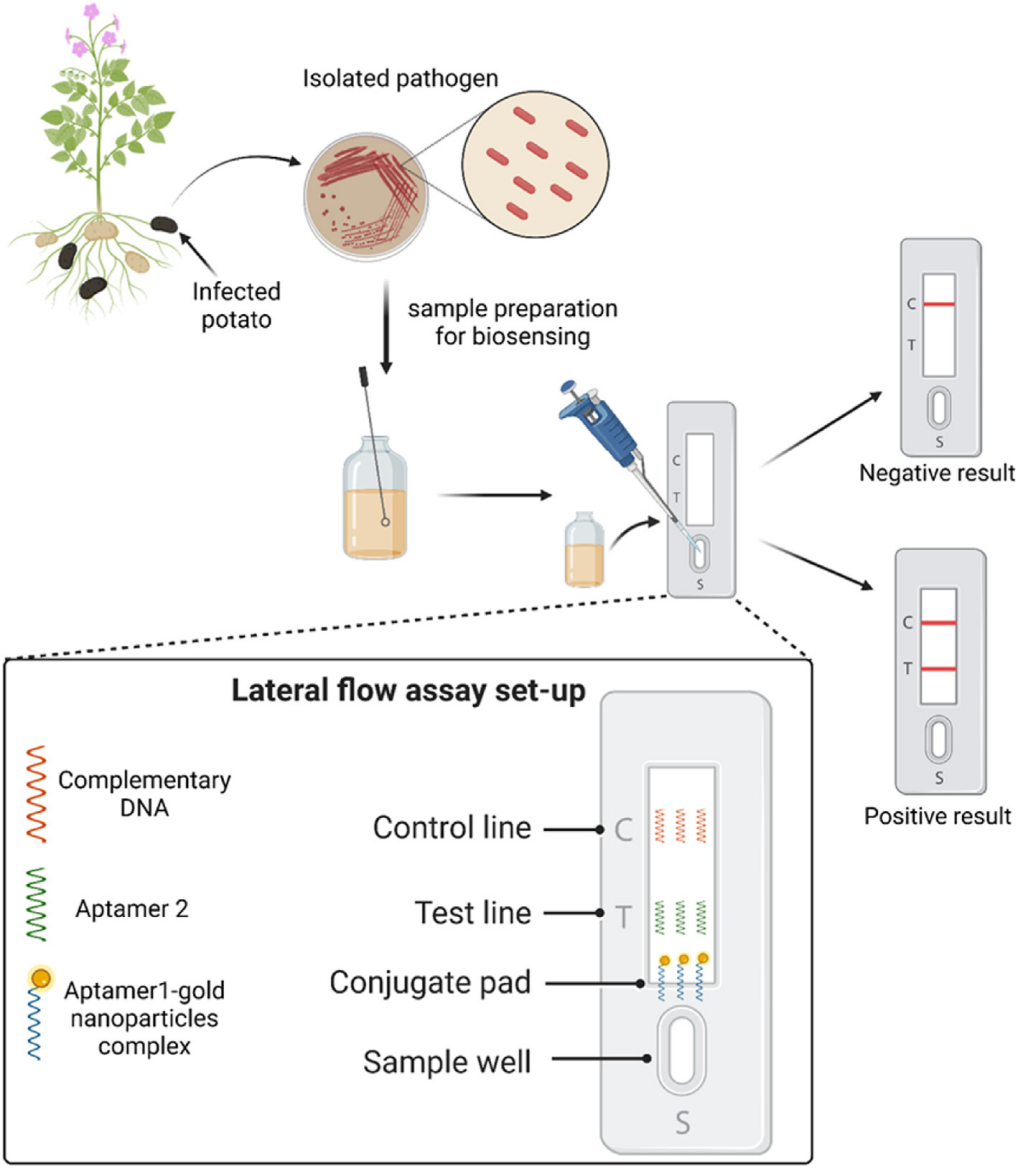
Lateral flow assay mediated detection of plant pathogen.

Plant pathogens are the prime reason for less crop productivity and may result in food scarcity for both humans and animals. It is predicted that food production will need to increase by 35–50% from 2012 to 2050 to feed the world population (∼10 billion people in 2050) (FAO 2019). It is estimated that globally 16% of crop yield losses happen due to plant pathogens (Ficke et al., 2018). One devastating example is the Irish Potato Famine or Great Irish Famine from 1845 to 1849 caused due to the late blight disease of potato (Goss et al., 2014). *Phytophthora infestans* are the causal agent of late blight disease. Along with this, some other plant pathogens are also present such as *Acidovorax avenae*, *Pseudomonas syringe*, *Ralstonia solanacearum*, *Pantoea stewartii*, *Botrytis cinerea*, *Pseudocercospora fijiensis*, *Leptosphaeria maculans*, etc (Dyussembayev et al., 2021). Most of the studies on plant pathology aim to reduce catastrophic crop loss directly or indirectly (Li et al., 2020). Plant diseases can be controlled by foliar as well as soil application of various pesticides, insecticides, and fungicides. But overuse of these toxic chemicals has some detrimental ecological issues and long-term use of this may develop some resistance properties among the pathogens. Hence the development of simple and rapid sensing tools or platforms has been key to research areas in plant disease management. In modern agriculture, nanomaterial-based biosensors are being used as a rapid detection tool. Researchers have developed different biosensing strategies such as electrochemical sensing, immunosensing, aptasensing, etc. Cebula et al. (2019) developed a label-free electrochemical (impedimetric immunosensor) biosensor to detect the *Pseudomonas syringae pv. lachrymans* (*Psl*) with a LOD of 337 CFU/mL. In this assay, a 4-aminothiophenol-glutaraldehyde-anti-Psl antibody-modified gold electrode was used (Table 2). In addition, another electrochemical sensor was present to detect the *Pseudomonas syringae* DC3000 (Psy) with a LOD of 214 pM (Lau et al., 2017). The detection process depends on the isothermal amplification of DNA sequences (target pathogen) by recombinase polymerase amplification (RPA) followed by the electrochemical assessment (Table 3). Vaseghi et al. (2013) developed another colorimetric detection of *Pseudomonas syringae* through thiol-linked DNA-Gold nanoparticle probes. The detection process relies on the change of color (red to purple) during the detection process, which confirms the positive result. For the identification of the soil bacteria *Ralstonia solanacearum* (potato wilt disease-causing pathogen), a similar colorimetric-based detection method was also developed. In this assay no prior DNA amplification was required; the pathogen can be directly detected from the soil sample (Khaledian et al., 2017). Lau et al. (2016) designed a versatile surface-enhanced Raman scattering (SERS)-RPA-based point-of-care detection method, where multiple plant pathogens (like *Pseudomonas syringae*, *Botrytis cinerea*, *Fusarium oxysporum*) can be easily detected (Lau et al., 2016). *Pseudomonas aeruginosa*, a well-known human opportunistic pathogen also shows some biotic stress like seed germination blocking of Arabidopsis, root pathogenicity of Arabidopsis, and sweet basil (Chahtane et al., 2018; Walker et al., 2004). This versatile pathogen can easily detect by a rapid, on-site detection process like aptamer-AuNPs-based lateral flow assay (Soundy and Day 2017) (Figure 2). Along with this researchers also developed a gold nanoparticles-based lateral flow assay to detect the fungi (*Phytophthora infestans*) and virus (Potato leafroll virus) (Zhan et al., 2018; Panferov et al., 2018).

**Table 2:** Different types of nanobiosensor and their role in agriculture.

Sl No.	Type of nanobiosensor	Nanomaterial used	Detection process/platform	Specific target	Limit of detection	References
Pathogen detection						
1	Chemiresistive	SWCNTs	Field-effect transistor-based detection of Sec-delivered effector 1, a secreted protein biomarker of HLB	*Citrus Huanglongbing* (HLB)	5 nM	Tran et al. (2020)
2	Colorimetric	Cysteine-functionalized AuNPs or AuNRs	Smartphone-based VOC-sensing platform	*Phytophthora infestans*	∼0.4ppm	Li et al. (2019)
		AuNPs	AuNPs-chimeric phages (M13) based detection platform	*Xanthomonas campestris*	∼100 cells	Peng and Chen (2018)
3	Electrochemical	Magnetic nanoparticles	DNA-based sandwich type detection system	*Phytophthora palmivora* (Butler) Butler (causing black pod rot in cacao (*Theobroma cacao* L.) pods)	0.30 ng DNA/μL	Franco et al. (2019)
		AuNPs	Antibody-modified gold electrodes based cyclic voltammetric detection platform	*Pseudomonas Syringae* pv. *Lachrymans*	337 CFU/mL	Cebula et al. (2019)
		AuNPs	Electrodeposited AuNPs modified sensing platform based on a screen-printed carbon electrode (SPCE)	*Citrus tristeza virus* (CTV) (causative agent of tristeza in citrus)	100 nM	Khater et al. (2019)
4	Lateral flow assay	AuNPs	Asymmetric ​PCR mediated *P. ​infestans* specific DNA probe-AuNPs based sensing assay	*Phytophthora infestans* (causing potato late blight)	0.1 pg μL^−1^	Zhan et al. (2018)
		AuNPs	AuNPs-streptavidin conjugate; Mouse anti-Fam antibody and BSA-Biotin conjugate based single-tube nested PCR-lateral flow biosensor assay (STNPCR-LFBA)	*Alternaria panax* Whetz	0.01 pg	Wei et al. (2018)
		AuNPs	AuNPs-Anti-PLRV antibodies-based sandwich type immunoassay	Potato leafroll virus (PLRV)	0.2 ng/mL	Panferov et al. (2018)
		CNPs	CNPs-polyclonal antibodies-based immunoassay	*Xanthomonas arboricola* pv. *pruni* (causal agent of the bacterial spot disease of stone fruits and almond)	10^4^ CFU/ml	Lopez-Soriano et al. (2017)
5	Localized surface plasmon resonance	AuNPs	AuNPs-DNA probe-based sensing platform	*Tomato yellow leaf curl virus* (TYLCV)	5 ng/μL	Razmi et al. (2019)
**Physiological stress detection**						
6	Electrochemical	AuNPs	Plant cell-based immune biosensor	Detection of vitronectin-like proteins (VN) a biomarker present on the surface of plant cells during heavy metals (cadmium or lead) stress	18.5 nmol/L for cadmium; 25.6 nmol/L for lead	Wang et al. (2019)
		Au ​nanostructures, ​Pt nanoparticles, ​reduced graphene oxide ​nanocomposite films	Disposable stainless steel-based ​microsensing platform	Detection of IAA under salt stress	43 pg mL^−1^	Li et al. (2019)
		MWCNTs	Differential pulse voltammetry-based detection process	Detection of salicylic acid and IAA	0.1 μM	Sun et al. (2015)
**7**	Localized surface plasmon resonance	AuNPs	Aptamer-functionalized AuNPs based detection process	Detection of abscisic acid (ABA)	0.33 μM	Wang et al. (2017)
8	Optical	AuNPs	AuNPs-oligonucleotide based sensing platform	Determination of the concentration of miRNAs as an indicator of drought stress	2 fM	Vakilian (2019)
**Toxic chemical detection**						
9	Colorimetric	AuNPs	DNA aptamer-AuNPs based colorimetric detection	Detection of λ-cyhalothrin	0.0197 μg/ml; 0.0186 μg/ml	Yang et al. (2021)
		AuNPs	DNA aptamer-AuNPs based colorimetric detection	Detection of tebuconazole	4.13 nM	Xie et al. (2021)
		AuNPs	Silk fibroin-gold nanocomposite-based AChE label-free bio-interfacial colorimetric method	Detection of chlorpyrifos	10 ppb	Mane et al. (2020)
		AuNRs	Based on inhibiting alkaline phosphatase (ALP)-induced silver metallization on the surface of AuNRs	Detection of Omethoate	0.53 U/L	Zhang et al. (2020)
		AuNPs	Tween 20 modified AuNPs based portable detection device	Detection of cyromazine	0.04 mg kg^−1^	Liu et al. (2020)
		AuNPs	Horseradish peroxidase + hydrogen peroxide + tyramine-induced AuNPs based direct competitive plasmonic enzyme-linked immunosorbent assay (dc-pELISA)	Detection of ochratoxin A	17.8 pg/mL	Liang et al. (2018)
10	Colorimetric/electrochemical dual-channel detection method	AuNPs	Aptamer modified Fe_3_O_4_@Au magnetic beads and cDNA modified AuNPs (cDNA-AuNPs) based sensing platform	Detection of aflatoxin B1	35 pg/mL and 0.43 pg/mL for colorimetric and electrochemical channel	Qian et al. (2020)
11	Electrochemical	Gold-graphene quantum dot nanohybrid	Arginine and aspartic acid-functionalized graphene quantum dot- gold-DNA aptamer based voltammetric sensing	Detection of omethoate and acetamiprid	1.67 × 10–14 Μ for omethoate; 3.33 × 10–15 M for acetamiprid	Ruiyi et al. (2022)
		AuNPs	Aptamer-AuNPs-SBA-15-Met (3D mesoporous structures) based detection model	Detection of bisphenol A	3.65 pM	Nodehi et al. (2021)
		AuNPs	Aptamer-AuNPs-polyaniline film-graphite screen-printed electrode-based voltammetric sensing	Detection of deoxynivalenol (mycotoxin)	3.2 ng/mL	Subak et al. (2021)
		AuNPs	Aptamers-AuNPs and conductive boron-doped diamond electrode-based sensing platform	Detection of aflatoxin B1	5.5 × 10^−14^ mol/L	Feng et al. (2020)
		Graphene nanosheets	Aptamers-positively charged poly (diallyl dimethylammonium chloride) modified graphene nanosheets and negatively charged carboxylated polystyrene nanospheres based sandwich type detection method	Detection of aflatoxin B1	0.002 ng/mL	Lin and Shen (2020)
		AuNPs	Monoclonal antibodies -AuNPs-modified screen-printed carbon electrodes based immunosensing platform	Detection of imidacloprid	22 pmol/L	Pérez-Fernández et al. (2020)
		AuNPs	Thiolated aptamers-AuNPs-differential pulse voltammetry-based label-free detection process	Detection of diazinon	0.0169 nM	Hassani et al. (2018)
		AuNPs	FTO-AuNPs and anti-chlorpyrifos antibodies (chl-Ab) based immunoassay	Detection of chlorpyrifos	10 fM	Talan et al. (2018)
		Platinum nanoparticles	PtNP microwires-Aptamers based impedimetric biosensing stage	Detection of acetamiprid and atrazine	1 pM for acetamiprid and 10 pM for atrazine	Madianos et al. (2018)
		Multi-walled carbon nanotubes (MWCNTs)/Dicyclohexyl phthalate (DCHP)	Acetylcholinesterase (AChE)-screen-printed electrode (SPE) based sensing platform	Detection of chlorpyrifos	0.05 μg/L	Chen et al. (2017)
		Graphene oxide	Aptamer-chitosan functionalized carbon black-graphene oxide@Fe_3_O_4_ nanocomposite-based sensing platform	Detection of chlorpyrifos	0.033 ng/mL	Jiao et al. (2017)
		AuNPs	Aptamer-AuNPs-polyaniline film-graphite screen-printed electrode-based voltammetric sensing	Detection of acetamiprid	0.086 μM	Rapini et al. (2016)
		Carbon nanotubes	Flake-like Fe_3_O_4_-carbon nanotubes-based Immunoassay	Detection of chlorpyrifos	6.3 pg/mL	Sun et al. (2015)
12	Fluorescence resonance energy transfer	AuNCs	AuNCs-Aptamer based sensing platform	Detection of AflatoxinB_1_ ​and Zearalenone	0.34 pg mL^−1^; 0.53 pg mL^−1^ respectively	Khan et al. (2019)
13	Lateral flow assay	Core-shell UCNPs	UCNPs-aptamer integrated smartphone-based portable sensing device	Detection of ochratoxin A	3 ng/mL	Jin et al. (2018)
		AuNPs	AuNPs-anti-triazophos monoclonal antibody based immunochromatographic assay	Detection of triazophos	250 ng/mL	Ge et al. (2020)
		AuNPs	AuNPs-monoclonal antibody based immunochromatographic assay	Detection for parathion, parathion-methyl, and fenitrothion	3.44, 3.98, and 12.49 ng/mL respectively	Zou et al. (2019)
		AuNPs	Monoclonal antibody-polydopamine-coated ​AuNPs based immunoassays	Detection of Zearalenone	7.4 pg/mL	Xu et al. (2019)
		Europium Nanosphere	Europium Nanosphere- monoclonal antibody-based time-resolved fluorescent immunochromatographic assay	Detection of aflatoxin B1, zearalenone and chlorothalonil	In maize: 0.16, 0.52, and 1.21 μg/kg respectively In peanut: 0.18, 0.57, and 1.47 μg/kg respectively	Wang et al. (2019)
		AuNPs	AuNPs-antibody based immunochromatographic strip test (ICST)	Detection of aflatoxin B1 and fumonisins	2 μg⋅kg^−1^; 1000 μg⋅kg^−1^ respectively	Di Nardo et al. (2017)
14	Photoelectrochemical	AuNPs	Aptamer-Poly (diphenylbutadiene)-BiOBr composite and AuNPs-linked CeO_2_ octahedron based photoelectrochemical sensing	Detection of acetamiprid	0.05 pM	Zheng et al. (2022)
15	Surface enhanced Raman scattering	AuNRs	AuNRs-nanoporous cellulose paper-based ​sensing platform	Detection of thiram, tricyclazole, and carbaryl	6, 60, and 600 ng ​cm^−2^ respectively	Kwon et al. (2019)
		Silver nanoparticle (AgNPs)	AgNPs assembled micro-bowl array	Detection of thiram and methyl parathion	1 fM	Zhu et al. (2020)
		AuNPs	Raman spectroscopy-based sensing platform	Detection of chlorpyrifos	1 μM	Xu et al. (2017)
		Silver colloid	Raman spectroscopy-based sensing platform	Detection of chlorpyrifos	10^−9^ mol/L	Ma et al. (2020)
**Heavy metal detection**						
16	Colorimetric	AuNPs	1-(3-(acetylthio) propyl) pyrazin-1-ium-AuNPs demonstrated a sensitive and selective spectrophotometric signal, as well as naked eye indication	Detection of palladium	4.23 μM	Anwar et al. (2018)
17	Electrochemical	SWCNTs	Vertical-SWCNTs) based cyclic voltammetric assay	Detection of mercury	3 fM	Shi et al. (2017)
18	Lateral flow assay	Core-shell UCNPs	UCNPs-aptamer integrated smartphone-based portable sensing device	Detection of mercury	5 ppb	Jin et al. (2018)
		AuNPs	AuNPs-streptavidin-biotinylated DNA probe based portable sensing device	Detection of mercury	2.53 nM	Guo et al. (2020)
		AuNSs	Competitive immunochromatographic strips based on the ability of AuNSs that quenching the signal of quantum dots	Detection of cadmium	0.18 ng/mL	Xiao et al. (2018)
19	Surface-enhanced Raman spectroscopy	AuNPs	AuNPs-trimercaptotriazine based ratiometric sensing	Detection of cadmium	8 μg/kg	Zuo et al. (2018)

**Table 3:** Oligonucleotide sequences against their specific target pathogen.

Target pathogen	Oligonucleotide sequences	References
*Phytophthora palmivora* (Butler) Butler	Detector probe: (5′- CGA ACA CTC CTC CAT TAA CGC CAC AGC AGA-3′); Capture probe: (5′- CCA CAA TCA GCA ACG CCA CGC TTT TGG AGC -3′)	Franco et al. (2019)
*Citrus tristeza virus* (CTV)	Thiolated ssDNA probe: 5‘- GGATCGATGTGTAA-3‘-(CH2)6‒HS; Target ssDNA (fully complementary; characteristic of CTV): 5’-TTACACATCGATCC -3’; Partially non complementary ssDNA (characteristic of Psorosis virus): 5′-TTACACAAGGATCT-3’; Fully non complementary ssDNA 5‘- TAGGATTAGCCGCATTCAGG-3’ as control sequence	Khater et al. (2019)
*Pseudomonas syringae* DC3000 (Psy)	5′-Forward-3′: TACACAGCAC(C3)TTTGTCCGAAACGACGTAC-AGCCATTTAACCTT 5′-Reverse-3′: Biotin-TTCTACGTCGGGGTATTTACTAGCTGG AAAAG Capture probe: GTGCTGTGTATTTTT-SH	Lau et al. (2017)

#### 7.1.2 Abiotic stress management

##### 7.1.2.1 Nano-biosensor-based monitoring of abiotic stress

**Figure 3:**
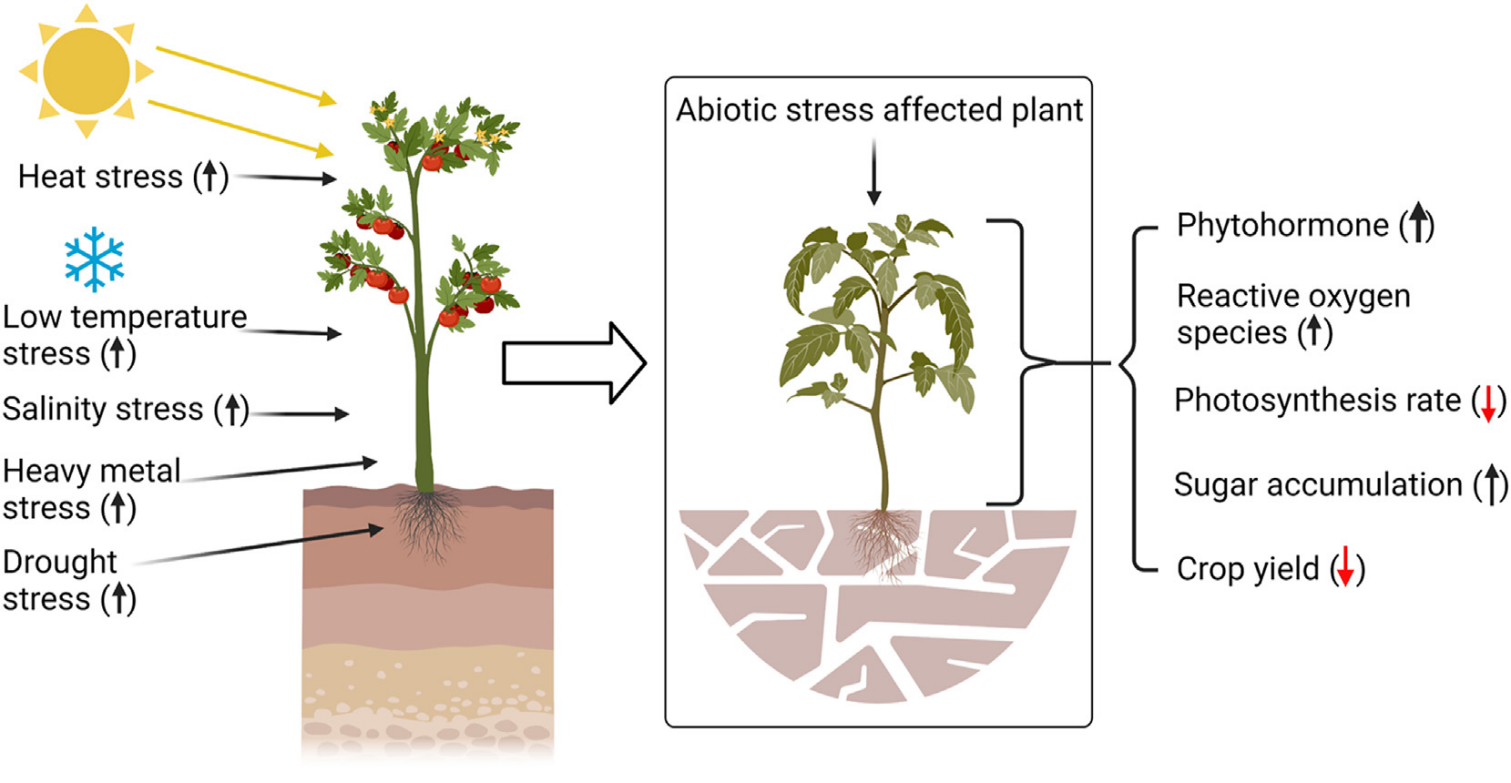
Different types of plant responses against the abiotic stress.

Plants are exposed to a wide range of environmental abiotic stress such as drought, salinity, light, heat, cold, heavy metals, and so on. These adverse conditions may alter plant physiologically, and biochemical responses e.g. changes in membrane composition, phytohormone responses, photosynthetic efficacy, sugar or other compounds accumulation, closure of stoma, production of small-molecules, and free-radicals, and activities of antioxidant enzymes and ultimately this will result in a drop in agricultural production (Husen 2022; Husen 2021a,b; Ansari et al., 2022) (Figure 3). Worldwide each year abiotic stress causes around 51–82 percent of crop loss (Li et al., 2021a, Li et al., 2021b; Oshunsanya et al., 2019). The Orissa famine of 1866 is a classic example of drought stress. To minimize abiotic-stress-mediated crop loss, it's essential to recognize stress and respond to it as soon as possible. Plants have different specific adaptive mechanisms to mitigate abiotic stress such as (i) restoration of cellular homeostasis, (ii) detoxification signaling to repair stress damages, etc (Kocabayet al., 2013). When plants are exposed to external stresses like saline, and water, heat the level of phytohormones like abscisic acid (ABA) (known as the plant stress hormone), indole-3-acetic acid (IAA) concentrations increase. Along with this, salicylic acid (SA), ethylene (ET), and jasmonates (JAs) have also specific activities under stress conditions (Mishra et al., 2016; Siddiqi and Husen 2019; Husen et al., 2018, 2019). Thus, researchers can monitor the plant abiotic stress responses through the detection of the phytohormones (Ansari et al., 2020). Hu et al. (2020) developed an electrochemical biosensor for the detection of IAA and SA from the soybean seedlings in presence of saline stress with a detection limit of 1.99 μM and 3.30 μM respectively. Multiwall carbon nanotubes (MWNT) and carbon black were used to increase the sensitivity of this detection system. Another electrochemical-based immune biosensor was developed to detect vitronectin-like proteins (VN). VN is a biomarker that is present on the plant cell surface under heavy metal stress. The detection limit of this system is 18.5 nmol/L for cadmium and 25.6 nmol/L for lead (Wang et al., 2019). Some other electrochemical biosensors were also present that can detect the phytohormones under different stress conditions (He et al., 2020; Wang et al., 2020; Cao et al., 2019). Surface plasmon resonance-based ABA detection was also developed with a limit of detection of 0.33 μM. This technique has relied on the AuNPs-aptamer conjugate (Wang et al., 2017).

##### 7.1.2.2 Current development in the application of nanomaterials in agriculture to mitigate abiotic stress

###### 7.1.2.2.1 Seed nanopriming

Plant seeds are an important part of agriculture; nevertheless, environmental factors (biotic and abiotic factors) can significantly influence seed germination and crop output during their early developmental phases. Stress (e.g. high salinity, drought, temperature, etc.) resistant seeds can result in increased agricultural output (Shelar et al., 2021). Seed priming is one of the prime approaches for maintaining cost-effectiveness, eco-friendly sustainable farming and offered different benefits including less amount of use of fertilizer and pesticides, high percentages of seed germination, and increased disease-resistant capacities in plants (Shelar et al., 2022; Mitra et al., 2021; Acharya et al., 2020). Nanoparticle-based seed priming (soaking of seeds in nanomaterials) influences seed germination and promotes plant growth by exhibiting different types of mechanisms such as the creation of nanopores for enhanced water uptake, generation of hydroxyl radicals for cell wall loosening, up-regulation of aquaporin genes and rebooting ROS/antioxidant systems in germinating seeds, etc. Furthermore, Nanopriming could boost α-amylase activity, resulting in greater soluble sugar content for supporting seedlings' growth (Mahakham et al., 2017). Kaffir lime leaf extract-based biosynthesized AgNPs can improve the starch metabolism and germination of aged rice seeds (Mahakham et al., 2017). Baz et al. (2020) reported that under high salinity stress conditions the carbon nanoparticles-based seed priming notably increases the seed vigor and seedling growth of lettuce by inhibiting the elongation of primary roots. Furthermore, another study showed that the zinc chitosan nanoparticles-based seed priming has antifungal activity. Seed nanopriming with zinc-chitosan nanoparticles enhanced seed germination and inhibited fungal growth in the case of maize crops (Choudhary et al., 2019).

###### 7.1.2.2.2 Nano-enabled plant genetic engineering

Nanoparticle-mediated transformation represents a promising approach for plant genetic engineering and may be a good substitute for gene gun bombardment, polyethylene glycol, or *Agrobacterium*-mediated transgenic process (Wu and Li 2022). Recent advancement in transgenic research has led to the development of high-yield stress-tolerant crops. A recent study showed that chitosan-complexed SWCNTs can be used in chloroplast-targeted transgene delivery and transient expression in plants. The SWCNTs selectively deliver plasmid DNA to chloroplasts via the lipid exchange envelope penetration mechanism (Kwak et al., 2019). For the assessment of the gene silencing mechanism, researchers developed different types of nanomaterials such as AuNPs, graphene oxide nanoparticles, DNA nanostructures, nanotubes, etc. to deliver siRNA into the mature plant tissues (Li et al., 2022; Demirer et al., 2020; Lei et al., 2020; Zhang et al., 2019).

###### 7.1.2.2.3 Nanoparticle-mediated targeted genome editing

Precise plant genome editing technologies have opened up new avenues for crop enhancement and the development of more sustainable agricultural systems. In particular, the CRISPR platform enables the precise editing of the crop genome and allows the development of high-yielding stress-tolerant crop variants (Sharma and Lew 2022). Nanomaterials have the potential to act as a platform for organelle-targeted CRISPR-Cas genome editing. Lv et al. (2020) have demonstrated gene editing in plants using magnetic nanoparticles.

### 7.2 Nanobiosensor in soil quality assessment

Since soil health is dynamic in nature, thus actual on-site monitoring of several soil parameters such as pH, nutrients, temperature, moisture content, etc. are crucial before any agricultural activities. Nanoparticles-based on-the-go measurements or sensor systems become indispensable for precision agriculture. In soil, a variety of nutrients exist in different forms, and each of the forms indicates different signals. For example, the presence of ammonia and high nitrate level in soil indicates the presence of soil-feeding insects and microbial imbalances respectively. A plethora of microorganisms are harbors in soil and greatly influence soil health and plant growth. Both ammonia and nitrate as well as nitrite are forms of nitrogen (Nettleton and Peterson 2011; Ji and Brune 2006). Researchers have developed versatile lipid-based nanobiosensors to detect the nitrites from the soil sample with a detection limit of 2.1 μg/L. This bilayer lipid membrane is held together through hydrophobic interactions and provides a mimicking environment for most of the elements for thermodynamic stability (Siontorou and Georgopoulos 2016).

Soil moisture content is another soil parameter to monitor and maintain crop yields. In the case of dry farming, Surya et al. (2020) developed an in-field rapid detection and quantification of soil moisture content based on an integrated capacitive sensor.

### 7.3 Nanobiosensor in toxic chemical detection

Chemicals in the form of pesticides, fungicides, insecticides, etc. are commonly used in agriculture to boost crop yields. These chemicals can persist in the applied area over a longer period. Apart from this, at a high dose, these chemicals can impose some acute detrimental toxicological effects on other living entities (Talari et al., 2021). Thus, their immediate and efficient monitoring is a crucial matter of concern. Traditional pesticide detection systems like chromatographic-based have various limitations like time consumption, the requirement of expensive equipment and highly trained technicians, sophisticated laboratory facilities, etc (Muenchen et al., 2016). As a result, the development of some rapid detection technologies is required that can easily detect this toxic chemical with a low detection time and limit. So far, numerous nano-biosensors (like electrochemical, colorimetric, lateral flow assay, etc.) have been developed for pesticide, insecticide, and fungicide detection (Table 2).

Talari et al. (2021) developed an aptasensor-based optical detection of diazinon (organophosphorus pesticides) with the LOD of 0.4 nM. The linear range of this sensor is 4–31nM. Multi-walled carbon nanotubes (MWCNTs), reduced graphene quantum dots (rGQDs), and fluorescent aptamers are used in this sensor. Talan et al. (2018) developed a fluorine-doped tin oxide (FTO)-AuNPs conjugated electrochemical immunoassay platform for the detection of the organophosphate pesticide viz. chlorpyrifos from vegetables and fruits with the LOD up to 10 fM. chl-Ab serves as a bioreceptor in this sensing platform. Another approach for the detection of organophosphate pesticides is fluorescent peptide probe-based detection. Here the peptides are attached with TPE, and in presence of pesticides, the peptide probe becomes aggregated to induce emission. Pesticides via covalent bonds react with the serine present in the peptide and cause aggregation. The LOD of this assay is 0.6 μM (Wang et al., 2020). Some enzyme-based biosensors like AChE, OPH, and OPAA-based biosensors have also been used to detect the organophosphate pesticide (Songa and Okonkwo 2016). Porto et al. (2022) developed a silver nanoparticle-carbon nanotube based-highly sensitive electrochemical sensor for the detection of multiple pesticides i.e. malathion (MLT), diazinon (DZN), and chlorpyrifos (CLPF), with the help of pyrolytic graphite electrode (PGE) from different sources like tap orange juice, water, and apple samples. The LOD of this sensor is 0.35 μmol/L for DZN, 0.89 μmol/L for MLT, and 0.53 μmol/L for CLPF. Fungicides like tricyclazole and tebuconazole can easily be detected by means of surface plasmon resonance and colorimetric-based sensing technology respectively (Xie et al., 2021; Kwon et al., 2019). Gold nanoparticles act as an indicator for both sensing platforms.

### 7.4 Nanobiosensor in heavy metal detection

The presence of heavy metals in higher concentrations in the environment has become a global threat (Mitra et al., 2022). Rapid amplification of heavy metals in the environment is due to the boost up of industrial, and mining activities like ore extractions, fuel emissions, improper industrial waste management, and usage of agrochemicals. Agriculture is the most affected area because of the use of agrochemicals in the form of herbicides, insecticides, fungicides pesticides, etc. that may release the metal ions into the field and then drain into the water bodies. Metal ions are carcinogenic, acquire a longer half-life, do not undergo biodegradation, and are bio-accumulative thus, showing a huge threat to human health (Ahmed et al., 2020). Several devastating events were related to heavy metal poisoning including the Minamata disease in Japan in 1956, the Itai-Itai disease in Japan in 1960, etc (Harada 1995; Nishijo et al., 2017). The presence of heavy metal ions induces oxidative stress in plants (Mathivanan 2021). Therefore, it is essential to detect the presence of heavy metals in the environmental sample. A variety of typical spectroscopic and chromatographic techniques are being used for heavy metals detection, but these techniques have some drawbacks such as it requires expensive sophisticated laboratory setups, trained laboratory personnel, and tedious sample preparation procedure, which makes them difficult to use as portable on-site easy-to-use detection systems (Eddaif et al., 2019). Thus, it is extremely important to accelerate the expansion of nanoparticle-based biosensors for on-site applications. Many serious attempts have been made to develop handheld or portable sensors for monitoring such heavy metals. The noble nanoparticles like AuNPs and carbon nanotubes helped to construct a rapid detection tool for mercury, cadmium, palladium, arsenic, etc (Jin et al., 2018; Xiao et al., 2018; Zuo et al., 2018; Anwar et al., 2018). Guo et al. (2020) developed a smartphone-based detection platform for Hg (II) as low as 2.53 nM. Lateral flow assay was another approach to detect mercury and cadmium by using UCNPs, AuNPs, aptamers, DNA probes, and antibody probes the detection limit of this assay was 5 ppb and 0.18 ng/mL respectively (Jin et al., 2018; Xiao et al., 2018). Lew et al. (2020) developed a near-infrared fluorescent nanosensor for real-time arsenic detection.

### 7.5 Smart nanobiosensors for crop health monitoring

Smart nanobiosensors, such as wearable sensors, have now emerged as a new frontier in plant and harmful chemical diagnosis and can pave the way for improved plant science research and agriculture. In the case of a wearable detection system, the sensors will be employed directly on the surfaces of different parts of the plant such as leaves and stems. Wearable sensors have attracted a lot of interest in diagnostics due to their low cost, flexibility, lightweight, and biocompatibility. Nassar et al. (2018) developed a stretchable strain sensor that assessed plant growth as well as temperature and humidity conditions of the plant leaf surface. Other smart plant leaf sensors like tape-based graphene sensors are present to monitor the plant's water transport behavior in real-time (Oren et al., 2017). Zhao et al. (2020) developed a laser-induced graphene technology-based plant-wearable biosensor for the electrochemical sensing of pesticides like methyl parathion. This detection process can be enhanced by the use of AuNPs and the detection output can be wirelessly transferred to the smartphone. Moreover, Li et al. (2021) developed a graphene-based plant volatile organic compounds (VOCs) detection during the plant biotic stress (tomato late blight disease) and abiotic stress (mechanical damage). This detection system can sense the VOCs release due to the stress within 4 days of tomato plant infection and 1 h after the mechanical damage. Kim et al. (2020) designed a polymer tattoo to sense the oxidative damage of fruits caused the excessive ozone exposure. In addition to this wearable plant sensor, e-monitoring devices such as electronic tongues (e-tongue), electronic nose (e-nose), and electronic eye (e-eye) have gotten a lot of interest in recent years for controlling and detecting plant pathogens (Mohammad-Razdari et al., 2022).

## 8 Future prospective and conclusion

The extensive use of nanobiosensors in the fashionable agri-food industries is accelerating gradually. The utilization of natural resources for improved productivity contributes to the sustainability of the agricultural and food sectors. These real-time nanobiosensors can be utilized to monitor different physiochemical properties of soil such as temperature, pH, soil moisture content, soil microenvironment, and nutrient status. Remarkably, these quick and reliable sensors have also been employed to detect residual pesticides, heavy metals, plant pathogens, fertilizers, and toxins which helps to predict and diminish crop losses in agroecosystems. To date, there are very few reports available for commercialized nanobiosensors in the agri-food industries. Modern nanosensor fabrication techniques include top-down and bottom-up approaches. In the top-down approach, electron-beam lithography, electrodeposition, fibre pulling, and chemical etching are reported to be the major fabrication techniques, whereas in bottom-up approaches nanosensors can be created using smaller components, pre-assembled in certain patterns which come together to form a finished sensor. Standard fabrication processes have been shown to be effective such as they can be performed in-house, precise and uniform-sized particles can be produced with cheaper and quicker fabrication processes. Nevertheless, a wide range of targets, including biomolecules, ions, and gases, can be easily detected. Further developments in the fabrication strategies may result in resource savings as well as increased performance. The dispersion of nanoparticles in terms of size, shape, and distribution is a limitation on existing fabrication procedures resulting in performance limits. However, cost-effective alternatives and the development of broad-spectrum nanosensors can boost their commercialization and remove the constraints related to their sensitivity and specificity. New analytical methods must be developed in order to recognize, evaluate, and access the impacts of each nanomaterial in the whole ecosystem. Size, dosage, exposure time, surface chemistry, structures, immunological response, accumulation, retention time, and other aspects of nanomaterials should be thoroughly examined for environmental impact assessment. The multidimensional potential of these customized and quick recognition systems should be explored further for their versatile, robust, and dynamic application in agroecosystems. Improving a broad databank as well as international collaboration in policy-making, strategy, and legislation are required to manipulate and disseminate such knowledge worldwide. Furthermore, clear standards and roadmaps for decreasing the dangers associated with the usage of nanotechnological goods should be provided by the concerned authorities. We hypothesized that with targeted efforts by governments and academia, nanotechnology will be transformational in the realm of agriculture by focusing research and development toward the aims of achieving sustainable agriculture.

## Declarations

### Author contribution statement

All authors listed have significantly contributed to the development and the writing of this article.

### Funding statement

Science and Engineering Research Board (SERB- File No. EEQ/2021/000058), Department of Science and Technology, Government of India financially assisted Amit Kumar Mandal through Empowerment and Equity Opportunities for Excellence in Science (EMEQ) scheme.

### Data availability statement

No data was used for the research described in the article.

### Declaration of interest's statement

The authors declare no conflict of interest.

### Additional information

No additional information is available for this paper.
